# Disseminated Juvenile Xanthogranuloma and Hemophagocytic Lymphohistiocytosis Developed During Treatment of Acute Lymphoblastic Leukemia: Case Report

**DOI:** 10.3389/fonc.2020.00921

**Published:** 2020-07-03

**Authors:** Katarzyna Pawińska-Wa̧sikowska, Magdalena Cwiklinska, Elzbieta Wyrobek, Walentyna Balwierz, Karolina Bukowska-Strakova, Agnieszka Dluzniewska, Jolanta Gozdzik, Grazyna Drabik, Monika Rygielska, Konrad Stepien, Szymon Skoczen

**Affiliations:** ^1^Department of Pediatric Oncology and Hematology, Faculty of Medicine, Jagiellonian University Medical College, Krakow, Poland; ^2^Department of Oncology and Hematology, University Children's Hospital of Krakow, Krakow, Poland; ^3^Department of Clinical Immunology and Transplantation, Institute of Pediatrics, Jagiellonian University Medical College, Krakow, Poland; ^4^Department of Clinical Immunology and Transplantology, Stem Cell Transplant Center, University Children's Hospital of Krakow, Jagiellonian University Medical College, Krakow, Poland; ^5^Department of Pathology, University Children's Hospital of Krakow, Krakow, Poland; ^6^Department of Biochemistry, University Children's Hospital of Krakow, Krakow, Poland; ^7^Student Scientific Group of Pediatric Oncology and Hematology, Jagiellonian University Medical College, Krakow, Poland

**Keywords:** acute lymphoblastic leukemia, non-Langerhans cell histiocytosis, hemophagocytic lymphohistiocytosis, juvenile xanthogranuloma, case report

## Abstract

The association between acute lymphoblastic leukemia (ALL), non-Langerhans cell histiocytosis (non-LCH), and hemophagocytic lymphohistiocytosis (HLH), to the best of our knowledge, has not been published to date. Juvenile xanthogranuloma (JXG), as a type of non-LCH, is usually a benign disease limited to the skin. Systemic involvement is rarely reported. The present case report describes a 15-year-old boy diagnosed with disseminated JXG involving skin and bone marrow concurrent with severe symptoms of HLH during ALL therapy. Examination of immunoglobulin heavy chain genes in B-cell precursor leukemic blasts and histiocytes in the skin and bone marrow revealed identical rearrangements, confirming clonal relationship between both diseases. Implementation of corticosteroids, vinblastine, etoposide, cyclosporine, and tocilizumab resulted in partial skin lesion resolution with no improvement of bone marrow function; therefore, hematopoietic stem cell transplantation (HSCT) was eventually performed. The patient's hematological and general status has improved gradually; however, remarkable recovery of skin lesions was observed after empirical antitubercular therapy. *Mycobacterium* spp. infection should be considered as a possible secondary HLH trigger. Triple association of ALL, non-LCH, and HLH highlights heterogeneity of histiocytic disorders and possible common origin of dendritic and lymphoid cells.

## Background

Histiocytoses are proliferative disorders of cells derived from dendritic cells or macrophages that occur predominantly in children. Recently, the Histiocyte Society prepared a revised classification system of histiocytoses, on the basis of clinical, genetic, and histologic features. In this classification, the histiocytic disorders are grouped into five categories: Langerhans-related (L), cutaneous and mucocutaneous non-Langerhans histiocytoses (C), malignant histiocytoses (M), Rosai–Dorfman disease (R), and hemophagocytic lymphohistiocytosis (HLH) and macrophage activation syndrome (H) (1).

Dendritic cells, monocytes, and macrophages belong to the mononuclear phagocyte system, whereas the term “histiocytes” refers to macrophages found in various tissues throughout the body (2).

Juvenile xanthogranuloma (JXG) is a benign proliferative histiocytic cell disorder of early childhood, and the most common form of non-Langerhans cell histiocytosis (non-LCH). JXG typically manifests in the skin as spontaneously regressing nodules, localized on the trunk, scalp, face, and extremities. Most patients with JXG have only cutaneous symptoms; rarely is extracutaneous manifestation observed, for example, bone marrow and bone involvement (3). JXG cases associated with leukemia are uncommon. There are single reports on JXG coexistence with juvenile myelomonocytic leukemia, T-cell and B-cell lymphoblastic leukemia, and acute monocytic leukemia, as well as neurofibromatosis type 1 (4–7).

HLH is a severe, life-threatening complication of infectious, rheumatoid, autoimmune, or malignant disease. It is caused by hyperreaction of T lymphocyte or macrophages, causing a cytokine storm manifested by fever, hepatosplenomegaly, lymphadenopathy, neurologic signs, or multiorgan failures (1, 8).

To the best of our knowledge, concomitant non-LCH with secondary HLH is unique in children being treated for acute lymphoblastic leukemia (ALL). Hence, we report on 15-year-old boy diagnosed with both disseminated JXG and severe secondary HLH with enteral enteropathy during the intensive treatment of ALL who was successfully treated with allogeneic hematopoietic stem cell transplantation (HSCT) followed by empirical antitubercular therapy.

## Case Presentation

A 15-year-old boy was referred to University Children's Hospital of Krakow, Department of Oncology and Hematology, in December 2016, because of progressive pallor, bone pain, and upper respiratory tract infection. Physical examination revealed general lymphadenopathy and no hepatosplenomegaly. At admission, severe anemia (hemoglobin level of 6.0 g/dl), leukopenia (white blood cell count of 3.4 × 10^9^/L), and thrombocytopenia (platelet count 45 × 10^9^/L) were found, prompting a bone marrow examination. Bone marrow biopsy revealed 81% of lymphoblasts presenting expression of CD45, CD19, CD22, and CD10; thus, the diagnosis was pre-B-cell precursor ALL (pre-B-ALL). A normal male karyotype (46, XY) was found in the bone marrow cytogenetic analysis. None of the prognostic genetic abnormalities such as *BCR-ABL, MLL-AF4*, and *ETV6-RUNX1* were found in leukemic cells. Cerebrospinal fluid examination revealed no lymphoblasts, and the patient was classified as CSN1 status.

The treatment according to ALL IC-BFM 2009 protocol was started, beginning with cytoreductive prednisone prophase and methotrexate intrathecally. Good response to glucocorticoids was seen. Induction therapy (Protocol IA) was continued afterwards with prednisolone, vincristine, and daunorubicin once per week with four total doses each and *Escherichia coli*-derived l-asparaginase with eight total doses and methotrexate intrathecally. Bone marrow aspiration on day 15 revealed no blasts (M1 bone marrow) and minimal residual disease (MRD) of 0.05%, indicating a good response to therapy. According to treatment protocol, the patient was ultimately stratified to intermediate risk group. Further treatment according to Protocol IB was continued: four blocks of cytarabine, cyclophosphamide, oral mercaptopurine, and intrathecal methotrexate. During the induction phase, apart from short-term pancytopenia, the patient did not present major toxicities of induction therapy. Complete remission with negative MRD (<0.01%) was achieved on day 78 (at the end of induction).

**Figure 1:**
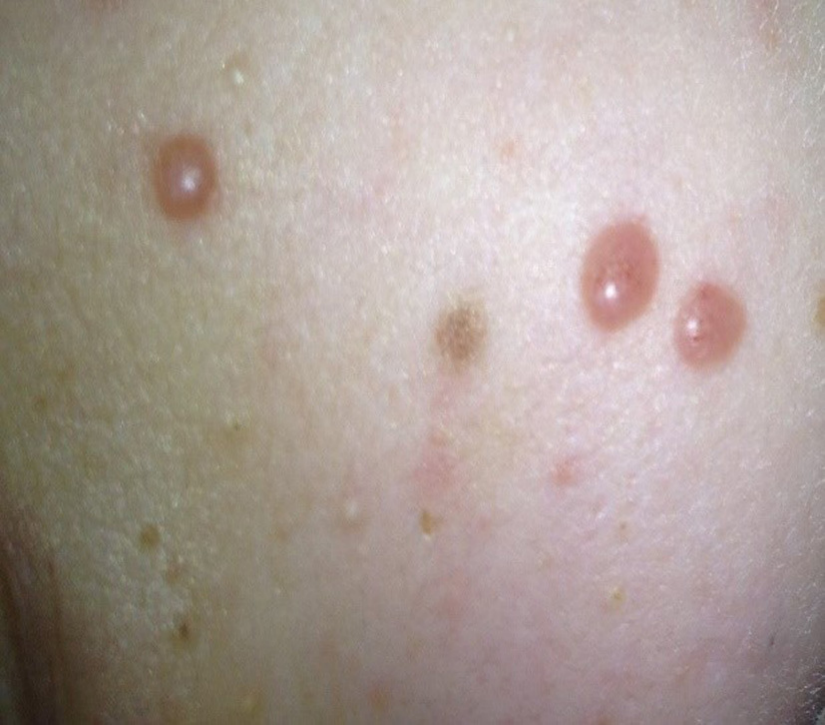
Multiple papulonodular skin lesions with predilection for cheeks.

**Figure 2:**
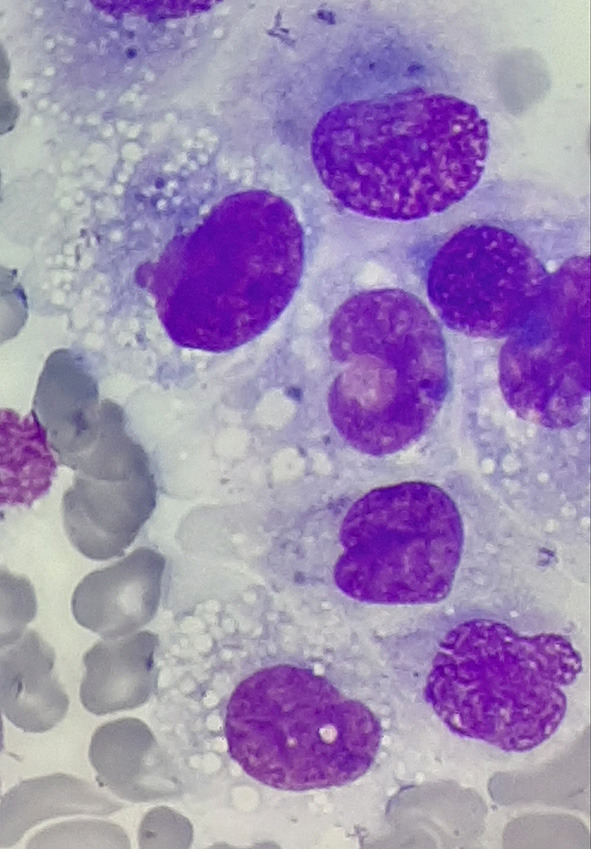
Histiocyte proliferation in the bone marrow of patient in remission of acute lymphoblastic leukemia (ALL); magnification, ×1,000.

**Figure 3:**
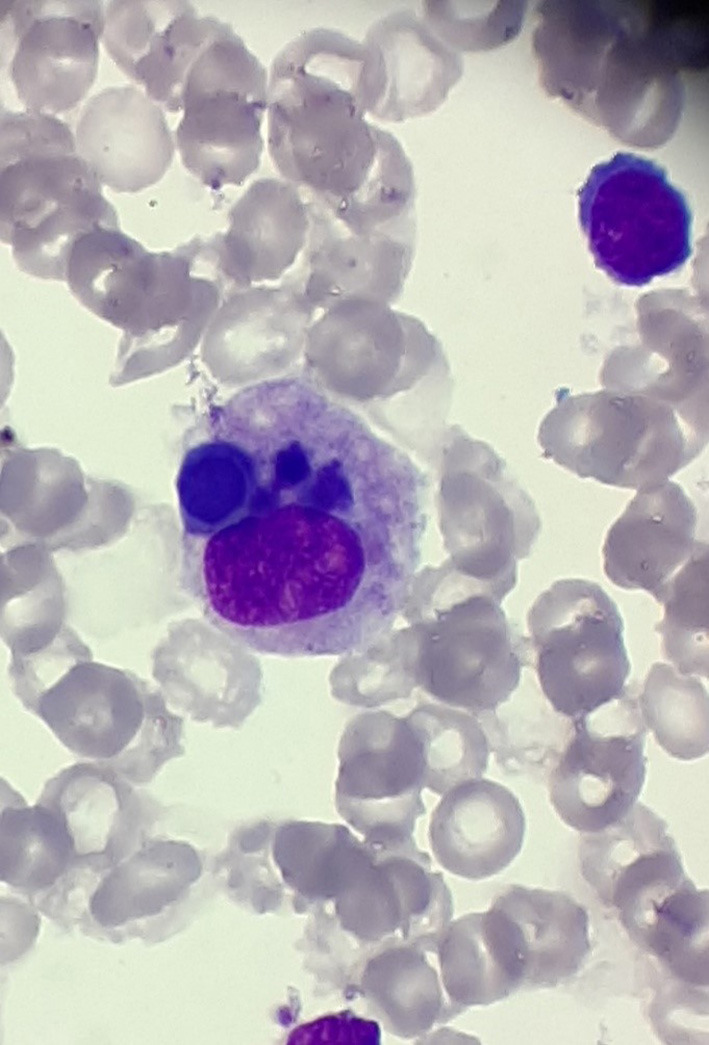
Hemophagocytosis in bone marrow; magnification, ×1,000.

At the beginning of consolidation, in March 2017, the patient was admitted to our department because of unexplained fever lasting 3 days. On physical examination at admission, disseminated, single, pearly yellow nodules localized on the face, trunk, and extremities (Figure 1); bruises; and significant hepatosplenomegaly were found. Complete blood count revealed severe pancytopenia with hemoglobin level of 6.0 g/dl, leukocytes count of 1.2 × 10^9^/L, and platelet count of 13 × 10^9^/L. Owing to suspicion of pending relapse, bone marrow biopsy was performed. Bone marrow examination confirmed persistent complete remission with negative MRD of <0.01%; however, an increased number of histiocytes with hemophagocytosis (Figures 2, 3) was seen in bone marrow. Flow cytometric analysis of bone marrow cells revealed abnormal population of cells presenting SSChigh and high expression of CD45, CD64, CD14, and CD33, evaluated as macrophages (16% of all nucleated cells). Bone marrow core biopsy was not performed owing to low platelet count and poor coagulation functions not responding to substitution treatment.

Because remission of ALL was confirmed, we started to explore other causes of pancytopenia. Firstly, the patient underwent infectious disease screening. Blood tests revealed elevated C-reactive protein (CRP) (44 mg/dl), with a low procalcitonin level. Blood and urine cultures, as well as viral panel testing for cytomegalovirus (CMV), Epstein–Barr virus (EBV), human herpes virus 6 (HHV6), HIV, parvovirus B19 (PVB19), hepatitis C virus (HCV), hepatitis B virus (HBV), hepatitis A virus (HAV), and herpes simplex virus (HSV), were negative, as were screening examinations for autoimmune and rheumatoid markers.

Within the next several days, the general status of the patient deteriorated, high-grade fever persisted, and hepatomegaly and splenomegaly progressed. Severe hypoproteinemia (30 g/L), hypoalbuminemia (16 g/L), and hypofibrinogenemia (0.6 g/L) was observed. Additionally, an elevated level of ferritin (1,850 μg/L) and CD25 level [soluble interleukin (IL)-2 receptor-−6,541 U/ml) were found. Cytotoxic tests of NK cells revealed their reduced activity (6%, normal range 9.9–26.1%). Finally, based on clinical manifestation (fever and splenomegaly) and laboratory tests (pancytopenia, hypofibrinogenemia, hemophagocytosis in bone marrow, high ferritin, and low activity of NK cells), the diagnosis was HLH according to HLH-2004 trial diagnostic criteria (9).

Considering the probability of secondary HLH with malignancy in the patient, extensive investigations for pathogens were carried out; however, none of the infectious triggers (bacterial, viral, fungal, or parasitic) were found at that time. In the diagnosis of primary HLH, defect in *SH2D1A* was excluded.

**Figure 4:**
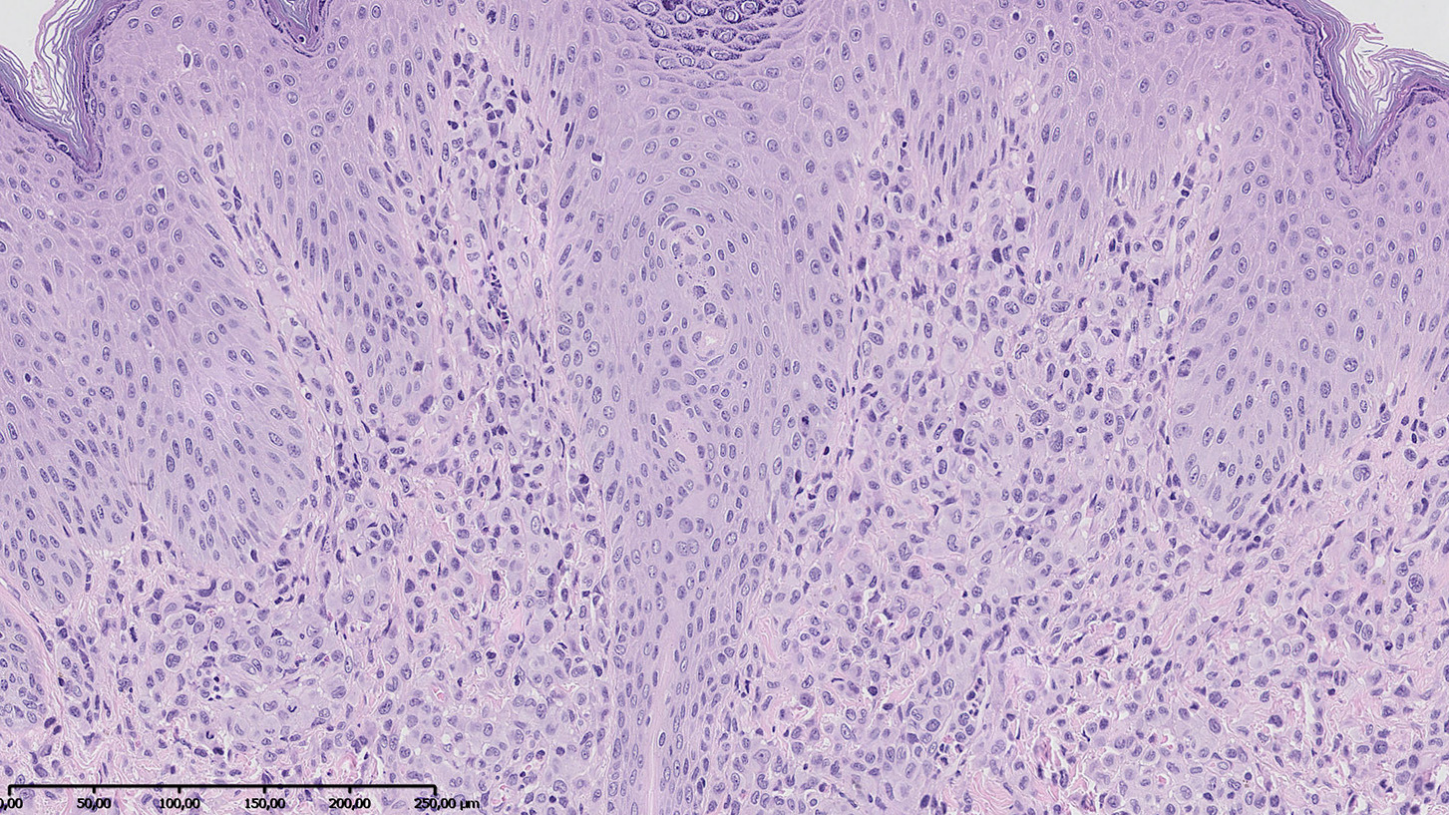
Skin biopsy revealed diffuse infiltration by histiocytes positive for CD45 and CD68 and negative for S100 and CD1a. Hematoxylin–eosin stain; magnification, ×10.

Additionally, owing to the appearance of new skin lesions on the face, trunk, and extremities of the patient, we decided to biopsy the nodules. Skin biopsy revealed diffuse infiltration by histiocytes expressing CD45 and CD68 but negative for CD1a, langerin, and S100 (Figure 4); thus, JXG was recognized. Furthermore, the DNA was extracted from the initial ALL bone marrow smears, with the bone marrow specimen showing histiocytic proliferation and current histiocytic lesions in the skin. The BIOMED-2-primer was used to amplify the immunoglobulin heavy chain gene. The PCR products were analyzed by capillary electrophoresis. There were predominant monoclonal amplifications of 305, 239, and 104 bp found in the bone marrow cells. Identical products were detectable in the histiocytic lesion of the skin, which finally proved clonal relationship between both diseases. No lymphoblasts were found in the skin biopsy.

Despite intensive supportive therapy, patient's status deteriorated gradually. Serious enteral enteropathy with extremely low protein (23 g/L) and albumin (11 g/L) levels resulted in considerable peripheral edemas and hydrothorax. Because the patient stayed in complete remission of ALL with negative MRD status, further therapy according to ALL IC-BFM 2009 was ceased, and treatment, based on HLH 2004 protocol, was introduced. At the beginning, methylprednisolone and etoposide were given; however, no improvement was seen. Thus, cyclosporine and vinblastine were added after 2 weeks. Owing to remarkable fluid retention, cyclosporine was stopped eventually. The patient still needed everyday transfusion of red blood cells, platelet, and plasma concentrates, as well as intravenous immunoglobulin supply. He was fed by gastric feeding tube and then by jejunal tube to fulfill caloric requirements.

In the beginning of April, the patient started to complain about bone pain of left scapula and upper extremities. MRI scan showed bone structure alterations of left clavicula, scapula, and humerus. However, biopsy of the lesion was not done owing to the poor general status of the patient, low platelet count, and persistent coagulation abnormalities.

Despite multidrug, intensive therapy, there was no improvement, neither in patient status nor in bone marrow function or skin lesions. Progression of hepatosplenomegaly was observed. Moreover, the patient developed additional side effects of steroids, such as diabetes, hypertension, and adrenal insufficiency.

Additionally, laboratory findings showed prominent pancytopenia, significantly elevated serum ferritin level, reduced fibrinogen level, and persistent hemophagocytosis in bone marrow. Looking for another treatment modalities of HLH, we decided to apply tocilizumab (IgG anti-IL-6 receptor). Once tocilizumab was given, a slow although unequivocal improvement of skin manifestation was seen. Increase of protein and fibrinogen levels, along with decrease of ferritin concentration, were achieved. Unfortunately, no change in blood parameters and features of hemophagocytosis in bone marrow were observed.

Finally, a total of five tocilizumab doses were given. Because there was no further improvement, the patient was planned to have HSCT. In August 2017, the allogeneic HSCT from matched sibling donor was performed. Patient received treosulfan, fludarabine, thiotepa, and alemtuzumab as a part of his conditioning. Since day 15, reconstitution of leukocyte count > 1 × 10^9^/L has been observed; neutrophils count > 0.5 × 10^9^/L since day 17; and platelet count > 50 × 10^9^/L since day 65 after HSCT. General and nutritional status of the patient gradually improved; however, some nodules on the skin of the face, trunk, and extremities were still present. One month after HSCT, the patient was discharged home. Ten days later, he was admitted to the hospital again owing to severe herpetic mucositis (WHO grade III). Because he started complaining of chest wall pain on the left side, X-ray was performed, and left side pleuritis was found. The pleura was punctured, and fluid examinations revealed high protein concentration and leukocytosis with presence of lymphocytes and macrophages. HHV6 PCR tests were positive, whereas aerobic and anaerobic bacterial, mycologic, and mycobacteria cultures, from both fluid and blood, were negative. *Mycobacterium tuberculosis* DNA by PCR test was not detected in pleural fluid.

Despite negativity of tests, still, atypical mycobacterial infection was suspected in our patient, as he had been treated with immunosuppressive therapy. Therefore, we decided to introduce antitubercular agents on an empirical basis. Once isoniazid and rifampicin were given, remarkable regression of all skin lesions was observed. Gradual increase of platelet number was also seen within the next weeks. The patient continued with antitubercular drug therapy for further 6 months. Currently, 2 years after HSCT, the patient's general health status is good, in continuous remission of ALL, HLH, and JXG.

## Discussion of the Underlying Pathophysiology and Significance of the Case

We report on a unique case of coexistence of disseminated JXG and HLH in a 15-year-old boy in remission of pre-B-ALL. According to our knowledge, there are several reports in the literature of concurrent existence of LCH and childhood leukemia. Rarely do patients with acute myeloid leukemia (AML) develop subsequent LCH. The more often pattern is ALL preceding the diagnosis of LCH and AML succeeding it. AML after LCH is probably secondary neoplasm triggered by chemotherapeutic agents used in LCH therapy (4–7, 10).

The association between ALL and histiocytic disorders has been investigated. Several articles have shown that LCH and ALL share the same mutations or had the same T-cell receptor or immunoglobulin rearrangement, which proves clonal relationship between them (8, 9, 11). Some papers report on *RAS* and *CDKN2A* mutations detected in ALL cases, which subsequently developed histiocytosis (8, 9).

Importantly, in our case, presence of identical genome alterations in both ALL and histiocytosis confirms that the latter is not the secondary malignancy caused by DNA damage owing to chemotherapeutic agents used for ALL treatment. Identical immunoglobulin heavy chain gene alterations detected in non-LCH and ALL, as well as short time of onset of histiocytosis (3 months) after initial ALL diagnosis in our patient, proves the common genetic origin in both. The association might be also related to suspected common progenitor cell defect of dendritic and lymphoid precursor (12). Unfortunately, we did not perform the whole exome sequencing (WES) analysis of B-ALL and non-LCH cells, which would be crucial for definitive estimation of clonal character of those two entities in the current case report.

For the future studies, the existence of common genetic background with specific molecular alterations brings opportunity for addition of molecular targeted therapies in patients usually resistant to conventional therapies (13, 14).

The association of ALL and HLH is more often reported, because secondary forms of HLH usually occur during different forms of immune system alterations, including malignancy, iatrogenic suppression induced by chemotherapeutic agents, HSCT, or infections (15).

In our patient, HLH could be malignancy or infection induced, even though we did not manage to prove infection trigger. HHV6 infection was documented, although it has been reported to be extremely rare as an HLH trigger (16). Moreover, HHV6 infection was detected late in our patient, after HSCT, and it is rather unlikely that it caused secondary HLH in this case.

The possible trigger of secondary HLH could be *Mycobacterium tuberculosis*. Tuberculosis is known as a great mimicker, owing to its diverse range of clinical manifestations, which make diagnosis difficult. Zhang et al. reported a study on adults with tuberculosis who had secondary HLH, with no underlying diseases. All patients manifested fever; additionally, half of them presented with skin rash, which was presented in different forms (17).

Furthermore, *M. tuberculosis* can induce Th1-mediated cytotoxicity, as well as can activate macrophages and NK cells, releasing more cytokines [IFN-γ, TNF-α, granulocyte-macrophage colony-stimulating factor (GM-CSF)], leading to tuberculosis and HLH symptoms (17, 18).

In HLH, excessive immune reaction leads to cytokine storm; thus, patients manifest a high level of IFN-γ, TNF-α, macrophage CSF (M-CSF), and ILs, including IL-1, IL-6, and IL-18. All those markers are responsible for diversity of clinical picture in HLH (15, 17–19).

Therefore, targeting specific cytokines might be an attractive therapeutic approach in HLH patients. Indeed, IL-6 is one of the contributors to the pathogenesis of HLH, which has been shown to induce defective expression of perforin and decreased NK cell cytotoxic activity (20, 21). Therapy with tocilizumab, targeting IL-6, helps to regulate the immune response in the course of HLH by diminishing inappropriate macrophage activation (20). The patient status improved after tocilizumab partially; however, he still presented with profound and persistent pancytopenia, which may suggest that IL-6 might not have a central role in the pathogenesis of HLH.

Moreover, ongoing ALL therapy, immunosuppression, and infections could induce overlapping histiocytes proliferation non-LCH and HLH in our patient. Triple association of ALL, non-LCH, and HLH highlights the heterogeneity of histiocytic disorders.

## Concluding Remarks

Diversity of non-Langerhans histiocytosis manifestation and severity of its course with systemic involvement in patient with malignancy make diagnosis and treatment highly challenging. Careful search for microorganism colonization/infection should be always performed especially in cases with concomitant HLH, considering mycobacterial infections as a possible trigger.

## Data Availability Statement

The datasets generated for this study are available on request to the corresponding author.

## Ethics Statement

Ethical review and approval was not required for the study on human participants in accordance with the local legislation and institutional requirements. Written informed consent to participate in this study was provided by the participants' legal guardian/next of kin. Written informed consent was obtained from the individual(s), and minor(s)' legal guardian/next of kin, for the publication of any potentially identifiable images or data included in this article.

## Author Contributions

KP-W, MC, EW, and SS contributed to case report concept and design. KP-W, MC, and KS wrote a section of the manuscript. KP-W, MC, EW, KB-S, AD, JG, GD, and MR performed diagnostic tests and collected relevant clinical data. WB, KS, and SS critically revised the article. All authors were responsible for the integrity and accuracy of the data and approved the submitted version.

## Conflict of Interest

The authors declare that the research was conducted in the absence of any commercial or financial relationships that could be construed as a potential conflict of interest.
